# Identifying Differences in the Performance of Machine Learning Models for Off-Targets Trained on Publicly Available and Proprietary Data Sets

**DOI:** 10.1021/acs.chemrestox.3c00042

**Published:** 2023-07-13

**Authors:** Aljoša Smajić, Iris Rami, Sergey Sosnin, Gerhard F. Ecker

**Affiliations:** Department of Pharmaceutical Sciences, University of Vienna, Josef-Holaubek-Platz 2, 1090 Vienna, Austria

## Abstract

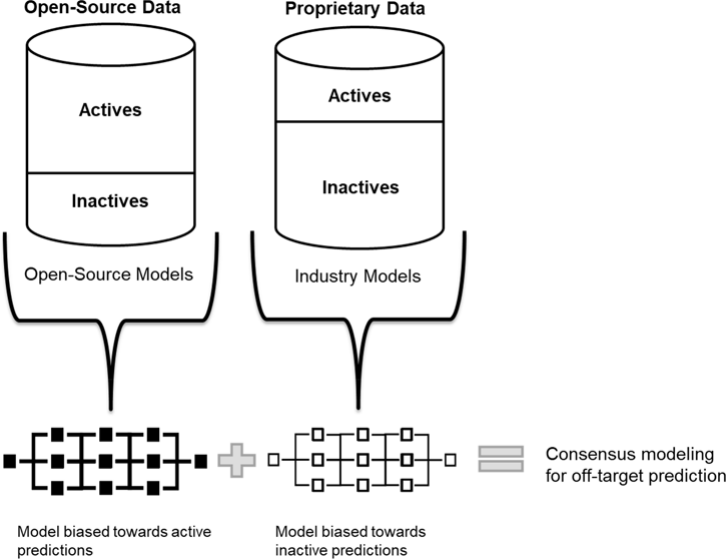

Each year, publicly available databases are updated with new compounds from different research institutions. Positive experimental outcomes are more likely to be reported; therefore, they account for a considerable fraction of these entries. Established publicly available databases such as ChEMBL allow researchers to use information without constrictions and create predictive tools for a broad spectrum of applications in the field of toxicology. Therefore, we investigated the distribution of positive and nonpositive entries within ChEMBL for a set of off-targets and its impact on the performance of classification models when applied to pharmaceutical industry data sets. Results indicate that models trained on publicly available data tend to overpredict positives, and models based on industry data sets predict negatives more often than those built using publicly available data sets. This is strengthened even further by the visualization of the prediction space for a set of 10,000 compounds, which makes it possible to identify regions in the chemical space where predictions converge. Finally, we highlight the utilization of these models for consensus modeling for potential adverse events prediction.

## Introduction

*In silico* toxicology has gained significant importance over recent years as the development of computational technology rapidly increases.^1,2^ The very recently signed FDA Modernization Act 2.0, which comprises a major shift away from animal use in drug development^3^ will further boost new approach methods in safety assessment. Quantitative structure–activity relationship (QSAR) is commonly used in *in silico* toxicology to predict the toxicity of chemicals based on their molecular structure. This statistical method requires data sets of chemicals and their known toxic effects for identifying relationships between the chemical structures of the compounds and their toxicities. The size of the data set and its quality have a decisive impact on the performance of the models.^4^ In scenarios where the data set is small or not representative of the broader chemical space, the model can fail to accurately predict the effect of new compounds. Many regulatory assessments, like the EU Registration, Evaluation, Authorization and Restriction of Chemicals (REACH)^5^ process, can be made possible with a greater knowledge of the uncertainties and limitations of QSAR modeling.

Combining several machine learning (ML) models to improve the final modeling performance is a well-known strategy in QSAR/QSPR studies.^6,7^ For example, Valsecchi et al.^8^ performed a large-scale case study on three properties and demonstrated that the consensus modeling generally outperforms individual models. The same conclusion was made by Khan et al.^9^ for ecotoxicity modeling for four different aquatic species. The authors applied the developed consensus models to DrugBank^10^ (https://go.drugbank.com) data to rank and prioritize potential ecotoxicants. However, the limitation of these studies was that all models were built on the same data set, varying only the methods used. Similar research was conducted in cooperation of several pharmaceutical companies in order to search for a new promising antimalarial therapy. This study revealed that model sharing in order to build a consensus can be a promising strategy. In this research, all models were trained on the basis of different proprietary data and then the performance of consensus modeling was estimated.^11,12^

While academic research institutions or government agencies often generate publicly available data, usually available in open-access databases such as ChEMBL,^13^ PubChem,^14^ or UniProt,^15^ pharmaceutical and biotech companies commonly have concerns about sharing their proprietary molecules with the public due to high investments related to the development of new drugs and other bioactive molecules. Based on the industry’s practices and the legal and financial implications of such companies, desired and informative knowledge about the chemical space may not be donated, even though it would be important for identifying potential off-target interactions or be used for other related approaches.^16^ However, a possible technique to share knowledge without providing confidential information is to use models built on proprietary data. In this way, information can be leveraged without disclosing individual compounds.

Nevertheless, as outlined in this paper, there is a fundamental difference in public and industry, especially in the off-target space. While in this case industry is optimizing toward inactivity, in the public domain there is a clear bias toward activity as (i) positive experiments are more likely to be published, and (ii) settings are more related to on-target effects, i.e., compounds are optimized for the respective target. This can have an impact on the resulting ML models and bias them toward the publicly available domain. Furthermore, proprietary data is often limited to the resulting series of substances from internal HTS campaigns, contrary to the public data that includes data by various experimental sources donated from different institutions and organizations.^17^

In this manuscript we created a series of off-target QSAR models from publicly available data sets and compared them with ML models from Roche’s in-house off-target optimized panel that followed the internal target selection methodology and assay protocol by Bendels et al.^18^ Using the ChEMBL database, four off-targets were selected for model building representing the public chemical space. The four targets, acetylcholinesterase (AChE), monoamine oxidase A (MAO-A), cyclooxygenase-2 (COX-2), and phosphodiesterase 4D (PDE4D), have been selected for model building due to the reasonable amount of data points within the publicly available database. To the best of our knowledge, this is the first study with detailed consideration of data bias related to private and public off-target data.

## Methods

### Data Set Preparation from ChEMBL

Data sets for targets described by Naga et al.^19^ were extracted from ChEMBL (ChEMBL30,^20^ ChEMBL31^21^), standardized and curated via the UNIVIE ChEMBL Retriever Jupyter Notebook (https://github.com/PharminfoVienna/cmpOffTargets). Only data points with human, single protein, and IC_50_ or *K*_i_ entries that shared the same gene name as in the work of Naga et al. were chosen for further curation and standardization steps. As shown in the literature, merging ChEMBL assays for the same target can be performed according to Kalliokoski^22^ and his colleagues. The IUPAC International Chemical Identifiers (InChIs), InChI keys, and simplified molecular input entry specification (SMILES) were calculated for each compound. As in the descriptor space used for model generation, stereochemistry is not considered, stereochemistry was removed from the InChIs to identify duplicates within the retrieved data sets. In the case of stereoisomers showing the same class label, one of the compounds was kept. Otherwise, both compounds were removed. MolVS (version 0.1.1) was used for compound standardization and cleaning, such as removing nonorganic compounds, salts, fragments, and charges. Following the same class labeling as that described by Naga et al., the pChEMBL threshold for active/inactive was set to 5. ChEMBL data sets for 41 out of the 50 off-targets were generated. Out of these targets, only four comprised more than 1000 compounds and a percentage of at least 15% of inactive compounds (see Table 1). In addition, a pChEMBL threshold of 6 was used for generating a second series of data sets which were also used for building ML models. This threshold was chosen based on previous research done by the “illuminating the druggable genome (IDG) group”, which has set activity thresholds based on target families (https://druggablegenome.net/ProteinFam).

**Table 1:** Data available for the Naga et al. targets on ChEMBL up to version 31, when using a threshold of pCHEMBL 5 and 6.

	Threshold = pCHEMBL 5			Threshold = pCHEMBL 6		
Target Name	Inactive	Active	% of inactive compounds	Inactive	Active	% of inactive compounds
Dopamine D2 receptor	205	6636	3.00	1505	5336	22.00
Acetylcholinesterase	951	3797	20.03	2356	2392	49.62
Serotonin 1a (5-HT1a) receptor	82	4267	1.89	553	3796	12.72
Serotonin 2a (5-HT2a) receptor	47	4273	1.09	707	3613	16.37
Mu opioid receptor	125	4157	2.92	945	3337	22.07
Adenosine A1 receptor	145	4025	3.48	1178	2992	28.25
Serotonin transporter	86	3949	2.13	783	3252	19.41
Adenosine A3 receptor	178	3805	4.47	925	3058	23.22
Histamine H3 receptor	84	3803	2.16	361	3526	9.29
Cannabinoid CB1 receptor	91	3776	2.35	958	2909	24.77
Kappa opioid receptor	133	3727	3.45	942	2918	24.40
Cyclooxygenase-2	573	2946	16.28	1646	1873	46.77
Glycogen synthase kinase-3 beta	292	3028	8.80	1146	2174	34.52
Estrogen receptor alpha	326	2246	12.67	826	1746	32.12
Matrix metalloproteinase 9	302	2169	12.22	679	1792	27.48
Monoamine oxidase A	987	1384	41.63	1800	571	75.92
Tyrosine-protein kinase ABL	159	2094	7.057	475	1778	21.08
Glucocorticoid receptor	28	2211	1.25	244	1995	10.90
Androgen Receptor	83	1969	4.04	580	1472	28.27
Cyclin-dependent kinase 2	180	1818	9.01	696	1302	34.83
Peroxisome proliferator-activated receptor gamma	96	1600	5.66	562	1134	33.14
Muscarinic acetylcholine receptor M1	129	1528	7.79	579	1078	34.94
Muscarinic acetylcholine receptor M2	120	1489	7.46	478	1131	29.71
Serotonin 2b (5-HT2b) receptor	13	1391	0.93	307	1097	21.87
Histamine H1 receptor	83	1299	6.01	389	993	28.15
Alpha-1a adrenergic receptor	8	1354	0.59	141	1221	10.35
Sodium/glucose cotransporter 2	40	1162	3.33	129	1073	10.73
Phosphodiesterase 4D	189	891	17.50	470	610	43.52
Dopamine D1 receptor	31	1041	2.89	294	778	27.43
Beta-2 adrenergic receptor	87	844	9.34	323	608	34.69
Alpha-2a adrenergic receptor	41	799	4.88	264	576	31.43
Beta-1 adrenergic receptor	96	711	11.90	385	422	47.71
Glycogen synthase kinase-3 alpha	55	742	6.90	304	493	38.14
Histamine H2 receptor	118	322	26.82	314	126	71.36
Cholecystokinin A receptor	10	389	2.51	86	313	21.55
Tyrosine-protein kinase ZAP-70	48	202	19.20	130	120	52.00
Voltage-gated L-type calcium channel alpha-1C subunit	48	112	30.00	111	49	69.38
Phosphodiesterase 3B	24	132	15.38	73	83	46.79
Neuronal acetylcholine receptor protein alpha-4 subunit	–	81	–	–	81	–
Angiotensin-converting enzyme 2	2	56	3.45	6	52	10.34
GABA receptor alpha-1 subunit	1	45	2.17	2	44	4.35

A time-series split for model validation was performed for creating and testing threshold 5 (TH5) models.^23^ For the training set, data up to the ChEMBL30 release was chosen. The newly released compounds in ChEMBL version 31 were identified and used for test set creation. In order to increase the size of inactive compounds for PDE4D, as only one compound was classified as inactive in the ChEMBL31 release, a different time series split was chosen. Thus, compounds up to the ChEMBL28^24^ release were used for training the ML model, and each compound added prospectively was used as a test set. The same test sets generated from ChEMBL were also used for evaluating ML models established by Naga et al.

Furthermore, all compounds up to ChEMBL version 31 were downloaded, filtered, and standardized using the UNIVIE ChEMBL Retriever Jupyter Notebook as described above. This complete ChEMBL31 data set was used for model training using the two thresholds pChEMBL value of 5 and 6.

### Data Set Preparation from Donated Pharmaceutical Data

For further validation of the models, data donated by pharmaceutical companies provided by the eTRANSAFE^25^ project were utilized. The data from eTRANSAFE were retrieved using Sirona, a knowledge hub developed by the eTRANSAFE project. The 4 off-targets were searched on eTRANSAFE, and only human single proteins were considered. These data sets were curated and standardized following the previously described pipeline. Furthermore, compounds already present in the ChEMBL training and test sets were removed from the eTRANSAFE data sets. In the eTRANSAFE data, the activity was mainly described in ‘percent inhibition’ at 10 μM, which corresponds to an activity threshold of pChEMBL value 5. Additionally, we calculated the distribution plots indicating the distributions of Tanimoto similarities of the test compounds (ChEMBL31 and eTRANSAFE) to the nearest train compounds for identifying if a potential test-set leakage is present. The Tanimoto similarities were calculated using ECFP descriptors (*r* = 3, bits = 4096). The resulting calculations can be found in the Supporting Information and indicate no leakage between the data sets.

### Data Set Preparation from DrugBank Data

The data set was downloaded as an XML file,^10^ and the necessary information (such as SMILES, compound name, UNII and CAS identification) was extracted using XPath nodes in KNIME. The retrieved data set was filtered using InChIs in order to exclude the ChEMBL data already used for training and testing the models as well as compounds with a molecular weight smaller than 61 Da and bigger than 2323 Da, which corresponds to the molecular weight range of the ChEMBL training and test sets. The retrieved data from DrugBank were further curated and standardized using the UNIVIE ChEMBL Retriever Jupyter Notebook.

### Statistical Metrics

To estimate the performance of the binary classification models and to put them into context with those from Naga et al.,^19^ the following parameters were used:

- Sensitivity: TP/(TP + FN)
- Specificity: TN/(TN + FP)
- Balanced accuracy: (sensitivity + specificity)/2
- Accuracy: (TP + TN)/(TP + FP + TN + FN)
- Precision or positive predictivity (PPV): TP/(TP + FP)
- Negative predictivity (NPV): TN/(TN + FN)
- Recall: TP/(TP + FN)
- F-Measure: 2 × ((precision × recall)/(precision + recall))
- Matthews correlation coefficient (MCC): (TP × TN – FP × FN)/sqrt((TP + FN)(TP + FP)(TN + FP)(TN + FN))

### Modeling Pipeline

**Figure 1:**
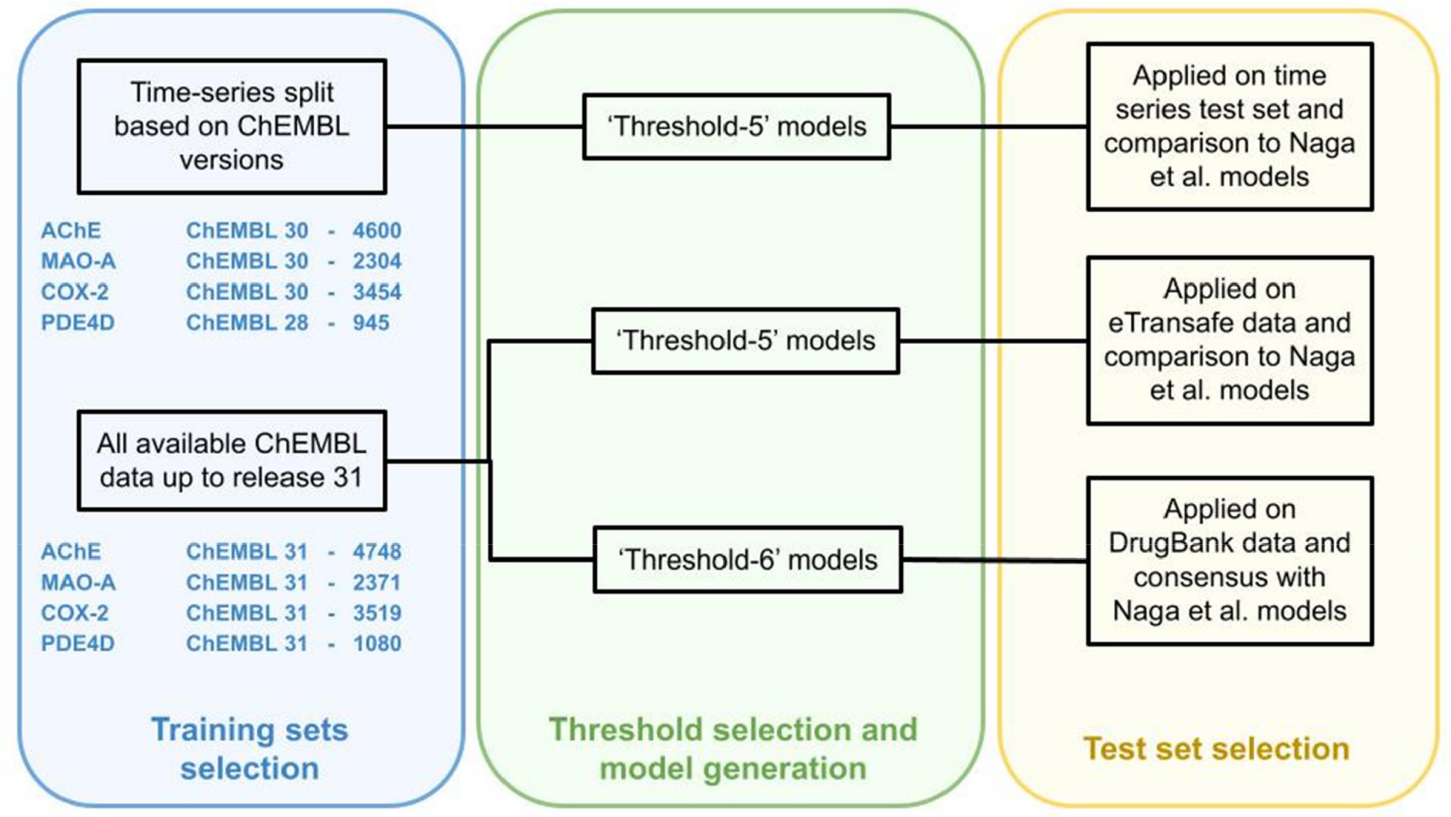
Depiction of the pipeline used to generate the models in this study. ChEMBL releases and the number of the compounds in corresponding releases are indicated in blue. For the time-series split models, ChEMBL30 was used as a training set for three off-targets and ChEMBL28 for the PDE4D model. For the other models, ChEMBL31, the latest version, was used. The green section indicates the chosen threshold (a pCHEMBL of 5 for the time-series split models and either pCHEMBL 5 or 6 for the other two models). In the yellow section, it is apparent that either the models were tested on the compounds of the remaining ChEMBL releases or on external data sets.

The pipeline used for the generation of ‘TH5’ models trained and tested using a time-series split as well as for the generation of ‘TH5 and TH6’ models built using all compounds available up to ChEMBL version 31 is depicted in Figure 1.

### Molecular Descriptors

In order to have a similar representation of the molecular descriptors as in the work of Naga et al.,^19^ extended connectivity fingerprint 4 (ECFP4)^26^ was chosen. The 1024 bits of ECFP4 were calculated for each compound from the standardized canonical SMILES using RDKit inside a Python Script KNIME node.

### Neural Network Architecture

**Figure 2:**
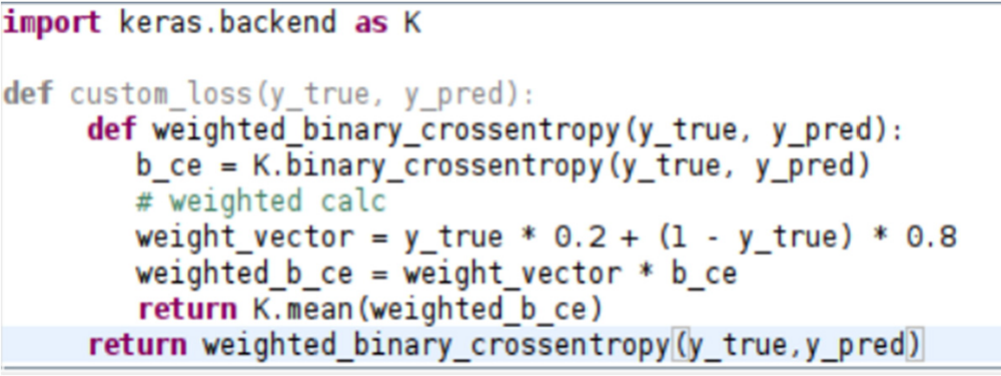
Applied weighted binary cross entropy.

We used one architecture for all experiments in this study. A feed-forward neural network was created using Tensorflow (version 1.12) and Keras (version 2.2.4) in KNIME (version 4.6.3). The general architecture of the neural network was created similarly to the work of Naga et al. The ECFP4 fingerprints were used as an input layer for the prediction of the binary activity of the compounds (active = 1 and inactive = 0). A sigmoid activation function was applied to the output layer, and the rectified linear unit (ReLu) function was used as an activation for the input and hidden layers. As the training set was imbalanced, the weighted binary cross entropy function was employed. Thus, a wrong prediction of the minority class leads to a greater loss than a wrong prediction of the majority class. The employed weighted binary cross entropy function is depicted in Figure 2.

Adam optimizer was employed with a learning rate of 0.01 to minimize the loss function. The learning rate decreased if the training loss showed no improvement over 10 epochs. Additionally, early stopping was employed if the training loss did not improve over 20 epochs. The neural network was trained and validated using an 80-20 training-validation split. Thus, 80% of the data was used for training the models, and 20% was used as a validation set for hyperparameter optimization.

### Hyperparameter Grid Search

A grid search over the hyperparameters was conducted via the parameter optimization loop start node integrated in KNIME (ver. 4.6.3) to identify optimal parameters for each off-target ML model. The numbers of hidden units, dropout rate and the training batch size used for tuning the hyperparameters are provided in Table 2. The hyperparameter grid search was used for each modeling experiment. The total grid search space resulted in 288 models.

**Table 2:** Hyperparameters Used in Grid Search.

	Start value	Stop value	Step size	Parameter
Hidden units	256	2048	256	Hidden units
Dropout input	0	0.2	0.1	Dropout input
Dropout hidden	0.2	0.4	0.1	Dropout hidden
Batch size	64	256	64	Batch size

The best hyperparameters were chosen based on the performance of the ML models on the 20% validation split. CSV files with the classification of each of the 288 models on the validation set were saved as the models were automatically built and applied to the validation set using the KNIME workflow.

As the output of the neural network is a value between 0 and 1, the classification threshold was set to 0.5 for classifying compounds as active (1) or inactive (0). A combination of the binary scorer node and the binary classification inspector node integrated into KNIME (ver. 4.6.3) was used to evaluate the performance of the generated models. Statistical metrics such as sensitivity, specificity, balanced accuracy, accuracy, precision, recall, f-score, and Matthews correlation coefficient (MCC) were calculated. Moreover, the MCC was used for selecting the best performing models bearing the optimal parameters, as MCC can be employed in imbalanced data sets and is suitable for binary classification evaluation.^27,28^

### Cross-Validation

A 5-fold cross-validation was performed applying only the best parameters and no grid search on each of the off-target models. This was done to ensure consistent results when using the best hyperparameters. The results for each of the 5 data splits were collected, and the binary scorer node on KNIME was used for evaluation. The metrics of choice in this case were the mean balanced accuracy, mean accuracy, mean recall, mean f-measure, and mean MCC as well as the respective standard deviation (SD).

### Training and Evaluation

For evaluation, different training and test set scenarios were used: First, ‘TH5’ models were generated based on a time series split as described in ‘Data set preparation from ChEMBL’ and using the general pipeline for generating the final models. The final models as well as the models from Naga et al. were applied to the time series ChEMBL test sets for performance check.

Second, all compounds up to ChEMBL release 31 were used for training the models again, keeping the pCHEMBL threshold of 5. These models were used on the eTRANSAFE data and on the DrugBank data set and were also used for model comparison and consensus scoring.

Finally, the activity threshold was changed to pChEMBL value of 6 and all compounds available up to ChEMBL version 31 were used for building this model. These models were used to assess the consensus scores with the Naga et al. models on the DrugBank data.

### Evaluation of DrugBank hits

Predictions were made on the DrugBank data set using the ChEMBL31 models. As ChEMBL31 ‘TH6’ models were specifically built in order to have a more balanced training set and to better reflect the positive data bias in public data sets, consensus classifications from Naga et al. models and ‘TH6’ models were analyzed. The analysis was done by searching for bioactivities on ChEMBL, by performing manual literature search on SciFinder and by using the pAERS database of adverse events^29^ (https://gpubox.cs.uni.edu).

The research on ChEMBL was done by drawing the query structure under ‘advanced search’ by pasting the standardized SMILES in the field. If the right structure was found, the bioactivities for the query structure were observed and the target of interest was searched by using the available filters. Furthermore, a manual literature search was performed by drawing the query structure on SciFinder and adding the target of interest to the search. Lastly, all positively predicted AChE inhibitors, which are FDA approved and have a trade name, were also researched in the pAERS database. In this database, adverse events are listed together with a *p*-value, which indicates the probability of the adverse event being connected to the drug. However, the drugs are listed only by their US trade names. AChE inhibitors were chosen as they are highly connected to specific side effects such as cholinergic crisis with well-studied salivation, lacrimation, urination, defecation, gastrointestinal distress, and emesis (SLUDGE) syndrome, plus miosis and muscular spasm (https://www.ncbi.nlm.nih.gov/books/NBK544336).

### Visualization of Prediction Space

The analysis of statistical parameters of models allows researchers to rank models or have a rough sense about models’ performance but does not provide a bird’s eye view of the chemical space that the model covers. Even worse, in the case of highly skewed data, the metrics can be misleading. To overcome these issues, we implemented a tool that allows visualization of the predictions of models across a given chemical space. For this, the predictions for four of our off-targets models (see Figure 4) and for the corresponding models from Naga et al.^19^ for 10000 compounds from the ChEMBL database were chosen. Uniform manifold approximation and projection (UMAP)^30^ was used as a visualization algorithm. UMAP projects molecular structures from a high-dimensional space (i.e., chemical descriptors space) to a 2D plane thus embedding the global structure of a high-dimensional space (space of ECFP fingerprints) into a low-dimensional Euclidean space. It is worth stressing that UMAP models were trained on molecular structures and have no information about any kind of activities. At the same time, given coordinates, one can visualize the predictions as a scatter plot using a color scale to represent any additional information layer. By utilizing this approach, one can select an off-target and choose between models based on public data and the models from Naga et al. trained on industry data.^19^ In Figure 4 a screenshot of the visualization tool is provided.

A set of visualizations of the same chemical space utilizing a custom distance function were also performed. The distance function regards not only structural similarity but also coherence of models’ predictions.
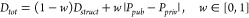
where *D*_*tot*_ is a distance function that is used in the UMAP algorithm, *D*_*struct*_ is the structural (Tanimoto) distance, *P*_*pub*_ is the probability by (our) public data driven model, *P*_*priv*_ is the probability by Naga et al. industry data driven model, and *w* is the coefficient that allows balance between structural similarity and the coherence of predictions. The UMAP model can follow either structural distance (when *w* is close to 0) or coherence of predictions (when *w* is close to 1), grouping compounds with similar predictions together. We introduced *w* because if we regard only structural distance, the UMAP projection can be biased toward structural moieties that provide a large variety of ECFP fingerprints (for example, adamantyl- group), ignoring structural features that are important for predictive models. Adding a tiny part of the distance in the prediction space pushed the model to focus more on groups that have an influence on the activity. It should be mentioned that normally, the value of *w* should be low, preserving well-formed clusters with visible and recognizable structural similarity. In our experiments, we revealed that *w* = 0.05 maintains chemical similarity while providing good grouping for compounds with similar predictions.

To quantify the performance of the visualizations in an objective manner we used the technique proposed by Karlov et al.^31^ We built a classifier on top of the 2D projections that has *X* and *Y* coordinates as input and tries to predict the original (in our case, “the original” means “predicted”) class at the output. Such a classifier can achieve any performance if, and only if, the overall distribution in the 2D space is meaningful (compounds within the same class grouped together). To eliminate our coherence contribution, we put w = 0 and rebuilt the UMAP. This resulted in a new projection that is entirely based on Tanimoto similarities. Subsequently, we trained an XGBoost classifier on top of the projections using 5-fold cross-validation and calculated the area under the ROC curve. To provide a null model, we shuffled the *X* and *Y* coordinates for the datapoints. To have the outputs equally distributed, we set the median of the predicted values as the thresholds for both ChEMBL and Naga et al. models.

## Results

### Data Set Overview

**Figure 3:**
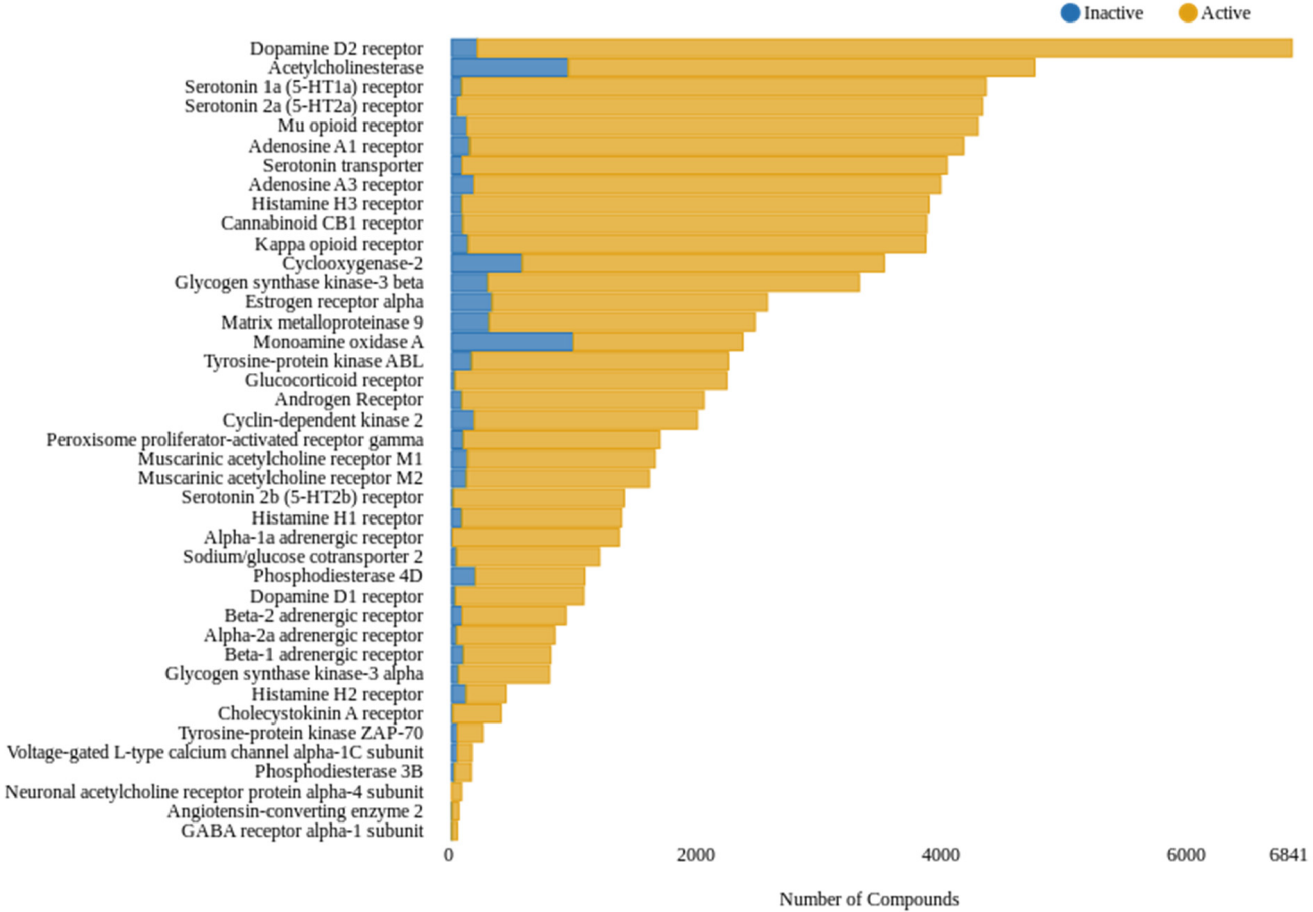
Overview of the total number of active and inactive compounds (pChEMBL threshold: <5) in the ChEMBL31 release. The *x*-axis represents the number of compounds found for each target in the ChEMBL database. The *y*-axis shows the selected panel of fifty off-targets. The color indicates the activity. Blue color indicates inactive compounds. Orange color indicates active compounds.

Simple statistical analysis was used for visualizing the number of active and inactive compounds for each target chosen from Roche’s in-house off-target optimized panel within the ChEMBL database. Figure 3 provides an overview of the distribution of active and inactive compounds within the publicly available domain by employing a pChEMBL threshold of 5. As can be seen, the size of active substances surpasses the size of inactive compounds in each of the selected targets. Notably, this is completely the opposite in the industry data sets used by Naga et al.

In order to keep the imbalance of the data sets as low as possible, we aimed to identify and select targets with a high coverage of inactive compounds within the retrieved data from ChEMBL31. Therefore, the percentage of inactive compounds represented in each data set was calculated. Only seven targets showed a percentage higher than 15 (Table 3). As can be further seen in Table 3, three of the seven targets had a low total sum of data points. For this reason, four targets (AChE, MAO-A, COX-2, PDE4D) have been selected for model building.

**Table 3:** Top seven off-targets sorted by the percentage of inactive compounds.

Off-targets	Inactive	Active	Sum	% of inactive compounds
Acetylcholinesterase	951	3797	4748	20.03
Cyclooxygenase-2	573	2946	3519	16.28
Monoamine oxidase A	987	1384	2371	41.63
Phosphodiesterase 4D	189	891	1080	17.50
Histamine H2 receptor	118	322	440	26.82
Tyrosine-protein kinase ZAP-70	48	202	250	19.20
Phosphodiesterase 3B	24	132	156	15.38

### Model Performance

The results of the 5-fold cross-validation are depicted in Tables 4 and 5. Each of the tables display the performance of the models by using different ChEMBL releases and thresholds for training. Since one of the tasks was the validation of models using data from the public domain, models were created and cross-validated based on compounds retrieved either from ChEMBL30 (AChE, MAO-A, COX-2) or from ChEMBL28 (PDE4). It is apparent from Table 4 that the performance of all models had a mean BA of over 70%, indicating good performances in terms of both the sensitivity and the specificity of its predictions. Also, the mean f-score in each of the models, with over 80%, shows that the models are able to accurately identify both positive and negative instances of the target class. Additionally, all four models were able to correctly predict the majority of the class labels for the data with a mean ACC over 75%. However, only AChE, MAO-A and PDE4D models had mean MCC values over 50% displaying a good balance of true positive (TP), true negative (TN), false positive (FP) and false negative (FN) predictions. Generally, all four models showed good performances and were used for external validation on publicly available data.

**Table 4:** Statistical Metric for All Four Off-Targets (pChEMBL TH5) Using 5-Fold Cross-Validation on ChEMBL30/ChEMBL28 Data^a^

	BA		ACC		Recall		F-score		MCC	
Off-target	Mean	SD	Mean	SD	Mean	SD	Mean	SD	Mean	SD
AChE	0.77	0.02	0.85	0.01	0.90	0.01	0.90	0.01	0.53	0.03
MAO-A	0.77	0.03	0.77	0.03	0.78	0.03	0.80	0.03	0.54	0.05
COX-2	0.72	0.02	0.85	0.02	0.92	0.02	0.91	0.01	0.46	0.05
PDE4D	0.85	0.03	0.91	0.01	0.94	0.02	0.94	0.01	0.69	0.04

a For each statistical metric, the mean value and the corresponding standard deviation (SD) is shown.

**Table 5:** Statistical Metric for All Four Off-Targets (pChEMBL TH5) Using 5-Fold Cross-Validation on ChEMBL31 Data^a^

	BA		ACC		Recall		F-score		MCC	
Off-target	Mean	SD	Mean	SD	Mean	SD	Mean	SD	Mean	SD
AChE	0.78	0.02	0.85	0.01	0.90	0.01	0.91	0.01	0.54	0.03
MAO-A	0.77	0.02	0.78	0.02	0.84	0.02	0.82	0.02	0.55	0.04
COX-2	0.71	0.02	0.86	0.01	0.93	0.01	0.92	0.01	0.45	0.05
PDE4D	0.84	0.08	0.90	0.04	0.93	0.02	0.94	0.02	0.66	0.13

a For each statistical metric the mean value and the corresponding standard deviation (SD) is shown.

Another aim was the validation of the models on data sets provided from pharmaceutical companies (eTRANSAFE data set). Therefore, our models were trained with all compounds up to ChEMBL31 release. The results of the cross-validation of the ML models are depicted in Table 5. Performances similar to those of the ChEMBL30/ChEMBL28 models have been observed in terms of BA, ACC, Recall, F-score, and MCC (see Table 5). The resulting models were then applied to the eTRANSAFE data set.

Finally, new models were created by increasing the pChEMBL threshold from 5 to 6 and the utilization of all compounds up to the ChEMBL31 release. Increasing the threshold to 6 (i) reduces the imbalance in the training sets and (ii) better reflects the bias toward active compounds in public data sources. The results of the 5-fold cross-validation are depicted in Table 6. One can see that the mean BA, mean ACC and mean F-score for three of the models indicated good performances with over 75%. Interestingly, a decrease of the mean f-score to 69% and mean recall to 65% can be observed in the case of MAO-A and a slight decrease for each other off-target of the mean recall from over 90% to a range from 82% to 88%. For the evaluation of the models, we mainly focused on the MCC results, as this metric is considered to be preferably used for unbalanced data sets.^27,28^ The mean MCC score for each off-target increased significantly for AChE from 54% to 68% and for COX-2 from 45% to 57%. In the case of MAO-A and PDE4D, slight increases of 4% and 1%, respectively, was obtained. These results suggest that the overall performance of the models has improved in terms of correctly classifying both positive and negative instances, leading to a better overall prediction performance.

**Table 6:** Statistical Metric for All Four Off-Targets (pChEMBL TH6) Using 5-Fold Cross-Validation on ChEMBL31 Data^a^

	BA		ACC		Recall		F-score		MCC	
Off-target	Mean	SD	Mean	SD	Mean	SD	Mean	SD	Mean	SD
AChE	0.84	0.01	0.84	0.01	0.86	0.02	0.84	0.01	0.68	0.03
MAO-A	0.79	0.02	0.86	0.02	0.65	0.04	0.69	0.04	0.59	0.05
COX-2	0.78	0.01	0.79	0.01	0.82	0.03	0.80	0.01	0.57	0.02
PDE4D	0.83	0.03	0.84	0.03	0.88	0.04	0.86	0.02	0.67	0.06

a For each statistical metric the mean value and the corresponding standard deviation (SD) is shown.

### Model Performance on Publicly Available and Pharmaceutical Test Sets

Tables 7 and 8 present the summary statistics for the performance of the ChEMBL30/28 models and models from Naga et al. tested on the ChEMBL31 test set. Table 7 illustrates that models built via ChEMBL data predict with higher reliability than those based on industry data (Naga et al., Table 8) when comparing the statistical metrics used to evaluate the performance. Interestingly, for the public models low specificity values from 27% to 33% can be observed for AChE and PDE4D, which indicates a high rate of false positives. An opposite scenario can be observed for MAO-A, where the sensitivity is 31%. When comparing the models, it becomes apparent that the models based on industry data are all biased toward predicting inactivity. This can be further displayed when the confusion matrices for each of the models.

**Table 7:** Statistical Metric for All Four Off-Targets Predicted on the ChEMBL31 Test Set Using ChEMBL for Model Training

Off-target	BA	ACC	Recall	PRC	Sen	Spe	F-score	AUC	MCC
AChE	0.56	0.65	0.79	0.73	0.79	0.33	0.76	0.70	0.13
MAO-A	0.61	0.60	0.31	0.79	0.31	0.91	0.45	0.73	0.27
COX-2	0.82	0.80	0.80	0.98	0.80	0.83	0.88	0.81	0.41
PDE4D	0.63	0.88	0.99	0.88	0.99	0.27	0.93	0.77	0.44

**Table 8:** Statistical Metric for All Four Off-Targets Predicted on the ChEMBL31 Test Set Using the Naga et al. Models Based on Industry Data

Off-target	BA	ACC	Recall	PRC	Sen	Spe	F-score	AUC	MCC
AChE	0.50	0.30	0	–	0	1	–	0.63	–
MAO-A	0.50	0.48	0	–	0	1	–	0.75	–
COX-2	0.51	0.11	0.02	1	0.02	1	0.03	0.75	0.04
PDE4D	0.50	0.16	0	–	0	1	–	0.19	–

Confusion matrices in Tables 9 and 10 show that models built on public data were able to predict a higher number of compounds as TPs than those based on industry data, which were unable to predict hardly any TPs in the test set derived from public data. Based on the number of FN it can be observed that industry models tend to overpredict inactive compounds when applied to the ChEMBL31 test set. Generally, all four test sets contain more active than inactive compounds. One can see in Tables 9 and 10 that the distribution of active and inactive compounds is highly imbalanced for AChE, COX-2 and PDE4D. Only for MAO-A a well balanced distribution with 35 active and 32 inactive compounds can be observed.

**Table 9:** Confusion Matrix Table Predicted on the ChEMBL31 Test Set Using ChEMBL for Model Training

	TP	FP	TN	FN	Total	Total P	Total N
AChE	81	30	15	22	148	103	45
MAO-A	11	3	29	24	67	35	32
COX-2	47	1	5	12	65	59	6
PDE4D	114	16	6	1	137	115	22

**Table 10:** Confusion Matrix Table Predicted on the ChEMBL31 Test Set Using the Naga et al. Industry Data Based Model

	TP	FP	TN	FN	Total	Total P	Total N
AChE	0	0	45	103	148	103	45
MAO-A	0	0	32	35	67	35	32
COX-2	1	0	6	58	65	59	6
PDE4D	0	0	22	115	137	115	22

The opposite behavior is seen when applying the models to the eTRANSAFE data set. When applying the models based on ChEMBL31 and a threshold of pChEMBL = 5 on the industry data-based test set, one can observe that models created by Naga et al. outperform the models created from public domain data. The performance of the public data-based models considerably decreased for AChE, MAO-A, and COX-2 when applied to the eTRANSAFE data sets.

Confusion matrices in Tables 11 and 12 show that retrained ChEMBL models were not able to predict TN correctly for AChE, MAO-A and COX-2, whereas models by Naga et al. correctly did. When models were applied to PDE4D one can see that most of the active compounds were correctly predicted by the public data-based model. Inactive compounds were better predicted by Naga et al. models. The results also indicate that models created from the publicly available domain tend to have a higher FP rate than the industry models when applied to the eTRANSAFE data. Taken together, these results suggest that models built on the publicly available domain classify data sets from industry incorrectly positive and tend toward a positive bias.

**Table 11:** Confusion Matrix Table Predicted from eTRANSAFE Data Using the ChEMBL31 TH5 Models

	TP	FP	TN	FN	Total	Total P	Total N
AChE	0	13	10	0	23	0	23
MAO-A	0	16	14	1	31	1	30
COX-2	1	18	5	2	26	3	23
PDE4D	8	2	2	0	12	8	4

**Table 12:** Confusion Matrix Table Predicted on eTRANSAFE Data Using the Naga et al. Models

	TP	FP	TN	FN	Total	Total P	Total N
AChE	0	1	22	0	23	0	23
MAO-A	1	0	30	0	31	1	30
COX-2	1	2	21	2	26	3	23
PDE4D	0	0	4	8	12	8	4

### Consensus Scoring for Risk Assessment

To exemplify potential use cases for the off-target models, we applied both the public and industry data derived models to DrugBank data. In the case of our public data models, we used the ones trained with a threshold of pChEMBL6 to reduce the bias toward predicting activity. If both our models and those of Naga et al. predict a compound as positive for a given off-target, we consider this a warning toward a potential risk. When applying the ChEMBL31 threshold pChEMBL 6 models and the industry data Naga et al. models on DrugBank data provided by Wishart et al.,^10^ only two out of the four off-targets showed consensus scoring when predicting compounds as active. For AChE, 29 compounds were predicted active by both models, and for COX-2, 143 compounds were classified as active. The remaining prediction results are as described in Table 13.

**Table 13:** Actives-Inactives Predictions of Our Models Trained with Threshold pChEMBL 6 and Naga et al. Models as well as Active-Inactive Consensus for Both Models

	ChEMBL31 TH6 Models		Naga et al. model		Consensus	
	Actives	Inactives	Actives	Inactives	Actives	Inactives
AChE	1855	8251	82	10024	29	8198
MAO-A	416	9690	13	10093	0	9677
COX-2	2790	7316	603	9503	143	6856
PDE4D	3020	7086	0	10106	0	7089

Unfortunately, a manual literature search on ChEMBL and SciFinder for bioactivities of the compounds predicted as active by both models for COX-2 was not successful. However, for AChE one hit was found on SciFinder. In the SciFinder abstract it is clearly stated that lomustine, which is an alkylating agent used in tumor therapy (https://meshb.nlm.nih.gov/record/ui?name=Lomustine), decreased AChE activity when administered to young and adult rats.

Furthermore, a search in pAERS database was performed on the predicted AChE inhibitors as described above. Here, two compounds were found. On the one hand, interesting results on the above-mentioned lomustine were found under the trade name Gleostine. Seizures were reported side effects for lomustine with a *p*-value of 0.00199. Even though there are reports of known AChE inhibitors potentially linked to seizures the actual correlation is questionable.^32^ On the other hand, plecanatide, a medication used for chronic idiopathic constipation (https://www.fda.gov/news-events/press-announcements/fda-approves-trulance-chronic-idiopathic-constipation), was found under the trade name Trulance to be linked to muscle spasms with a *p*-value of 1.0E-7 in pAERS. As there is a strong correlation between muscle spasms and AChE inhibition, this reported adverse effect might be an indication that plecanatide is actually linked to AChE inhibition.^33^

### Visualization of Prediction Space

**Figure 4:**
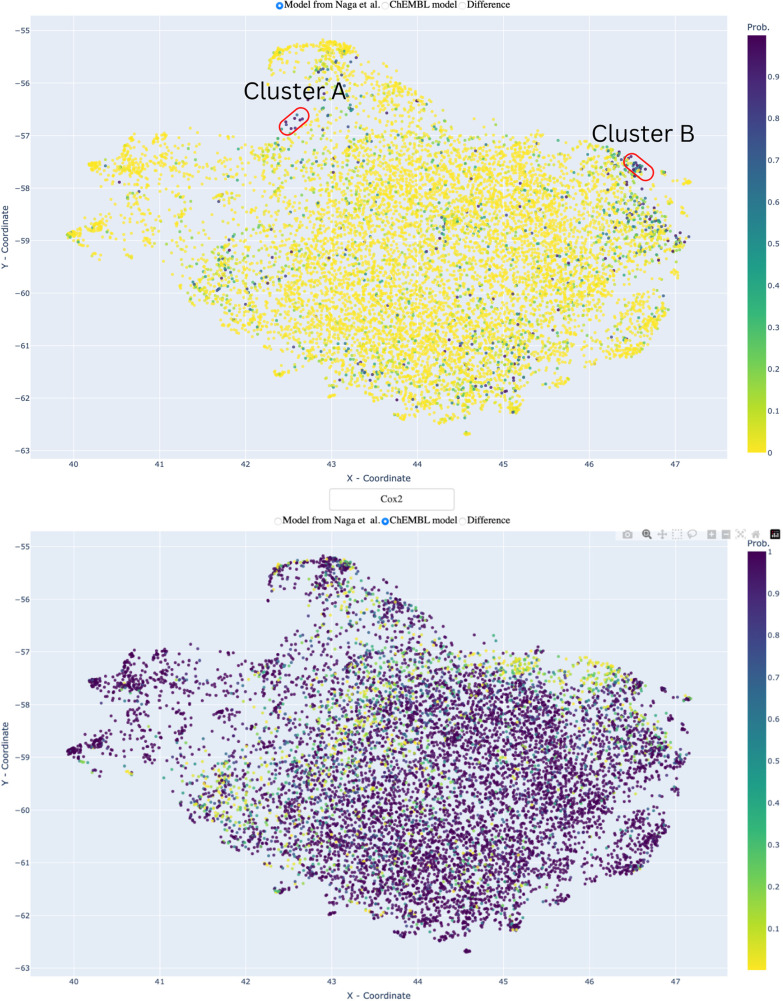
UMAP visualization of ChEMBL and Naga et al. Cox2 models’ prediction space for 10000 compounds from ChEMBL. Color represents the probability of being active. Yellow indicates that the probability is low; in contrast, pink indicates high probability. One can see that the ChEMBL public data model is heavily biased toward predicting compounds as active, while the Naga et al. industry data model is heavily biased toward predicting compounds as inactive.

**Figure 5:**
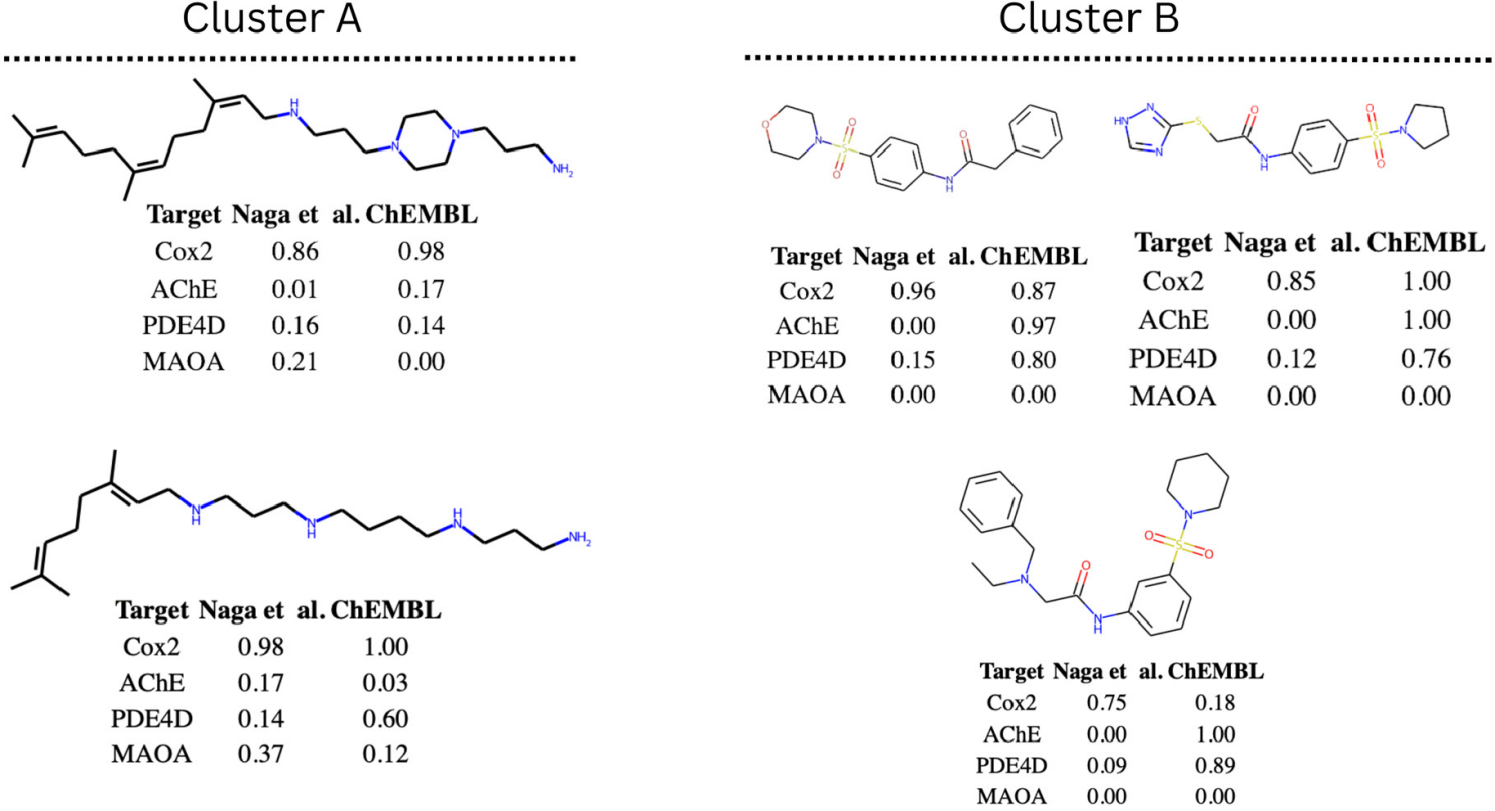
Visualization of typical compounds from two clusters of compounds that were predicted as being active by the Naga et al. model (see Figure 4). The right-hand cluster (B) mainly consists of sulfanilamide derivatives. The left-hand one (A) consists of aliphatic polyamines.

Visualizing the prediction pattern of the ChEMBL public data and the Naga et al. industry data models using the UMAP algorithm clearly demonstrates the models’ bias (Figure 4). Naga et al. models predict the majority of compounds to be inactive, while the compounds that were predicted as active are smoothly distributed across the plot. In contrast, models derived from ChEMBL data predict most of the compounds to be active, while inactive compounds form two distinguishable clusters. While typically the compounds predicted as actives by the Naga et al. industry data derived model are spread across the whole space and do not form clusters, we were able to identify two dense clusters comprising sulfonamide derivatives and aliphatic polyamines (see Figure 5), where for the off-target COX-2 both models (Naga et al. and ChEMBL) predicted activity with high probability. However, it should be noted that the ratio actives/inactives in the prediction space roughly resembles the respective distribution in the training set, with MAO-A showing the most balanced ratio (45%/55%). We provide our visualization tool freely available to the community on GitHub, so that the researchers themselves can investigate the prediction space of the models from this study. Also, we estimated the performance of our visualizations (see Methods section). The results are given in the Supporting Information (Table S3). One can see that the performance of XGBoost models vary from 0.62 to 0.69 ROC AUC. At the same time, the performance of the permuted models is about 0.5 as expected. So we can conclude that UMAP can provide a meaningful way for the projection of compounds into 2D space.

### Analysis of the Applicability Domains of the Models

**Figure 6:**
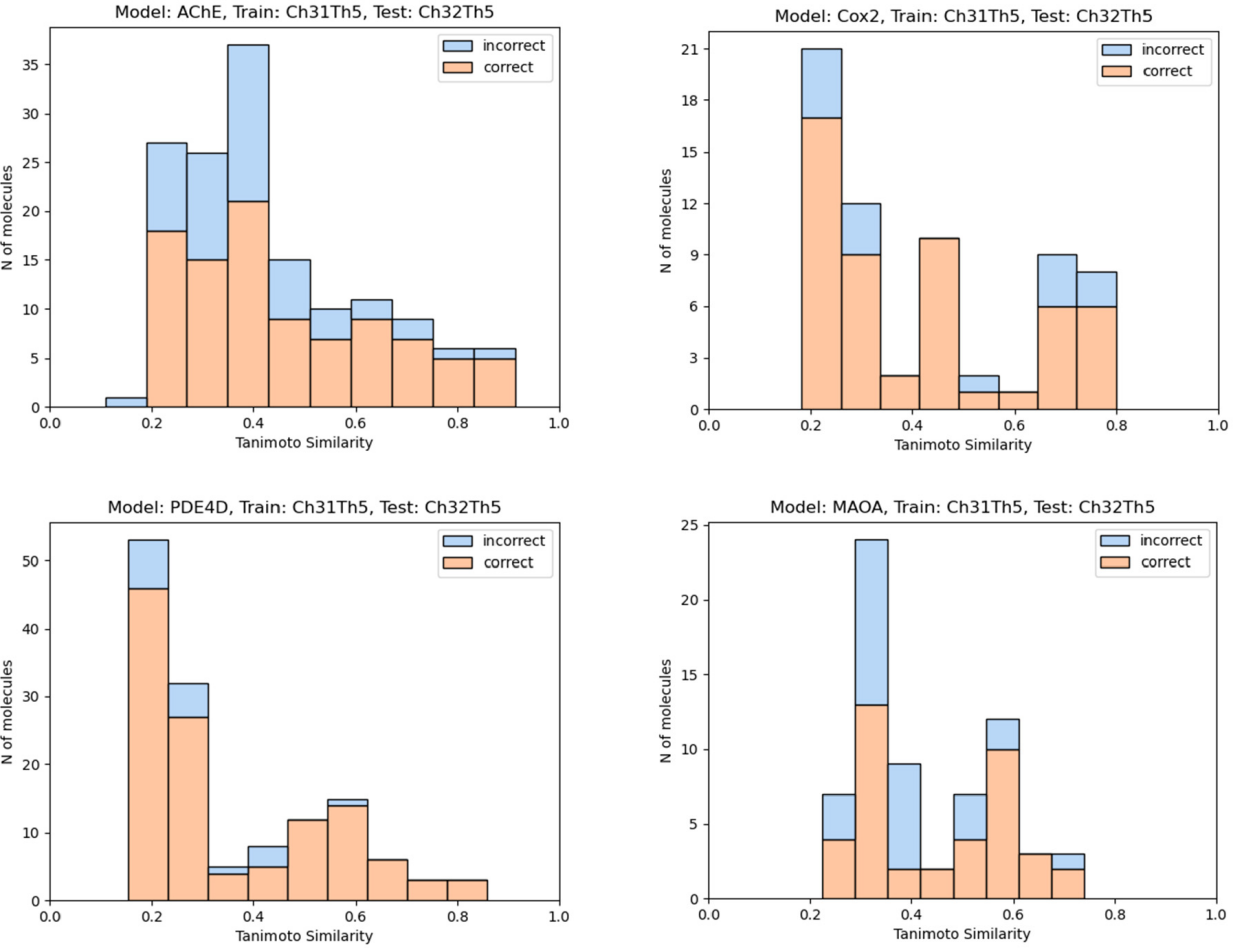
Tanimoto similarity distributions for the compounds from the test set to the nearest member of the training set. Blue, incorrect predictions; red, correct predictions.

To reveal the applicability domains of the models and indicate potential data leaks between the training and test sets, we performed the calculation of the Tanimoto Similarity for each compound from our test sets to the nearest member of the training set. We prepared a series of figures for our four models trained on ChEMBL30 and ChEMBL28 and tested on ChEMBL 31 (see Figure 6). Additionally, we conducted the same experiment for the ChEMBL31 and eTRANSAFE data. For Drugbank data, since we did not have access to ground truth values, we opted to plot only the histograms of Tanimoto similarities. The eTRANSAFE and Drugbank figures are provided in the Supporting Information (Figure S1). Figure 6 demonstrates that there are no indications of leakage between the training and test splits due to the absence of compounds with Tanimoto similarities equating to or nearly reaching one. Furthermore, there is a weak tendency for incorrect predictions for compounds far from the model, as measured by the Tanimoto similarity.

## Discussion

An initial objective of the project was to identify the influences of publicly available and proprietary data on the model training and its use for off-target prediction. One interesting finding is the distribution of positive and negative data entries within the publicly available and pharmaceutical industry domain. Table 1 and Figure 3 show that data derived from ChEMBL are biased toward active compounds when applying either standard off-target screening thresholds (pIC_50_ of 5) or IDG values. Since data sets extracted from ChEMBL are often used for model training in research,^34−36^ this can be an issue when applied these models in off-target oriented industry setups. By visualizing the distribution of active and inactive compounds in ChEMBL, a high imbalance toward active compounds can be observed (see Figure 3). This might be explained by the general setting of academic projects, which aim for producing active compounds. Furthermore, publishers still ask for positive results rather than accepting work which predominantly demonstrates inactivity of compound series. In consequence, this bias toward positive results renders the creation of statistically significant ML models (see Tables 7 and 8) complicated. Notably, only 4 out of 41 targets (AChE, COX-2, MAO-A, PDE4D) had both a percentage higher than 16 for inactive compounds at a threshold of pChEMBL 5 (see Table 3) and a data set size of more than 1000 compounds. In contrast, data sets derived from industry in-house off-target screens show an opposite picture.^19^ This poses the question if the high positive data bias present in ChEMBL (when thresholds for off-target screens are used) would bias the model creation toward the overprediction of active compounds, and vice versa for models derived from industry off-target screens. To verify this, models were created by using ChEMBL data for model training, and model performance was compared with models established by Naga et al., which used Roche internal off-target screening data for model building. For this comparison, both test sets derived from the public domain and from the pharmaceutical industry were used.

When comparing the performance of classification models from both domains, one can see that the models built from publicly available data tend to overpredict active compounds, whereas models built on industry data tend to overpredict inactive compounds (see Tables 7 and 10). By analyzing the performances on the ChEMBL31 test set, it becomes apparent that models by Naga et al. do not correctly identify any TP compounds except for one TP in the COX-2 test set. Interestingly, no compounds were predicted as FP. For some off-targets, the precision, F1-score and MCC do not have any values, as can be seen in Table 8. Some models do not predict any compounds as TP or FP (see Table 10) and a value of zero in both cases will result in a division by zero when calculating the precision; therefore, no value can be calculated for the precision. As both the F1-score and MCC are dependent on the precision, no values can be calculated for these two metrices either. An opposite observation of results can be displayed when models are tested on the eTRANSAFE data sets, where models by Naga et al. predict the majority of inactive compounds correctly (see Tables 11–13). Again, in cases in which no values are present, no TP and FP were classified by the models. Moreover, other metrices are missing for the AChE models as the eTRANSAFE data set does not contain any active compounds.

Models established on ChEMBL data predicted the majority of compounds as FPs, except in the case of PDE4D, where 8 out of 8 compounds were correctly classified as TPs and 2 out of 4 were predicted correctly as TNs. As models based on ChEMBL data were trained predominately on positive data, they tend to predict the majority class as positives (see Table 3). This supports the idea that off-target models based on ChEMBL data might lead to an overprediction of active compounds, and vice versa, the use of data from off-target HTS screens might lead to models overpredicting negatives. Therefore, a combination of the models for off-target modeling was used to create more robust predictions to identify potential risks related to interaction with off-targets. By screening DrugBank with both models based on public data derived with a less biasing activity threshold of pChEMBL 6 and those from Naga et al., only two out of four off-targets provided a set of compounds that showed consensus active labels. Extensive literature search indeed identified lomustine and plecanatide as potential AChE inhibitors.

Applying QSAR models to find chemical compounds with potentially adverse effects is a well-known scenario in *in silico* toxicology. However, our study highlights that positive and negative biases can occur in public and proprietary models. Consensus modeling may therefore be preferable in this situation. This could be demonstrated by identifying two established drugs, lomustine and plecanatide, which might be linked to AChE inhibition.

Visual analysis of the prediction landscape of all models gave a further indication of models’ bias. Although all models in our study demonstrate high performance in terms of statistical metrics, visualization of their prediction landscape for 10000 compounds reveals a clear bias but also areas in the chemical space where public and industry models converge.

Unbalanced data sets are a known challenge in the field of ML, and various methods have been developed to address it, such as data augmentation, data imputation, down-sampling or optimizing the Kappa statistic.^37^ None of the mentioned approaches were applied in this study as the main intention was to investigate a positive data bias in public data sources. However, for our models, we did shift the activity threshold value from 5 to 6 for pChEMBL to increase the stringency of the model when applied to assess the consensus scores with the Naga et al. models on the DrugBank data.

## Conclusions

In this study, we present a significant positive bias in models trained on public data and a negative bias for models trained on proprietary data. Additionally, we visualize the distribution of active and inactive compounds for 4 targets and point out the large imbalance between positive and negative entries in ChEMBL. Also, a visualization technique that allows the user to envision the respective prediction bias is offered. More importantly, this approach helped to demonstrate the importance of consensus modeling when models from different domains are used to predict adverse effects due to off-target inhibition. In this regard, we identified two compounds potentially related to AChE inhibition, plecanatide and lomustine. This finding further confirms the advantages of using consensus scores from models built on different domains when predicting off-target activity.

## Supporting Information Available

Tanimoto similarity distributions for the compounds from the test set to the nearest member of the training set for eTRANSAFE and Drugbank data, statistical metric for all four off-targets predicted on the eTRANSAFE, ROC AUC performance of the XGBoost classifiers built on top of UMAP visualizations (PDF)

## Abbreviations

Ch: ChEMBL
TH5: threshold 5
TH6: threshold 6
QSAR: quantitative structure–activity relationship
QSPR: quantitative structure property relationships
HTS: high-throughput screening
AChE: acetylcholinesterase
MAO-A: monoamine oxidase A
COX-2: cyclooxygenase-2
PDE4D: phosphodiesterase 4D
InChIs: IUPAC international chemical identifiers
SMILES: simplified molecular input entry specification
IDG: illuminating the druggable genome
CAS: Chemical Abstracts Service
CSV: comma-separated values
BA: balanced accuracy
ACC: accuracy
PRC: precision
Sen: sensitivity
Spec: specificity
AUC: area under the curve
MCC: Matthews correlation coefficient
TP: true positive
FP: false positive
FN: false negative
UMAP: uniform manifold approximation and projection for dimension reduction
SLUDGE: salivation, lacrimation, urination, defecation, gastrointestinal distress, and emesis
