# Supplementary material for: Identifying Differences in the Performance of Machine Learning Models for Off-Targets Trained on Publicly Available and Proprietary Data Sets
Source: Chem Res Toxicol. 2023 Jul 13;36(8):1300–12. doi: 10.1021/acs.chemrestox.3c00042 (PMC10445286; doi:10.1021/acs.chemrestox.3c00042)
Supplement: Supplementary file 1 — tx3c00042_si_001.pdf [file tx3c00042_si_001.pdf]

Supporting Information for Publication: “Identifying Differences in the Performance of Machine Learning Models for off-Targets trained on publicly available and proprietary datasets”

*Aljoša Smajić[#], Iris Rami[#], Sergey Sosnin, Gerhard F. Ecker**

University of Vienna, Department of Pharmaceutical Sciences, Josef-Holaubek-Platz 2, 1090 Vienna, Austria

[#] both authors contributed equally to the work

Off-Target Prediction, QSAR, Model Comparison, Consensus Modeling

Table of Contents

Supplementary Figures for the subsection “Analysis of the Applicability Domains of the models”............2

Tanimoto similarity distributions for the compounds from the test set to the nearest member of the training set for eTRANSAFE and Drugbank data 2

Supplementary Tables for the subsection “Results - Model Performance on Publicly Available and Pharmaceutical Test Sets” 3

Statistical metric for all four off-targets predicted on the eTRANSAFE..3

ROC AUC performance of the XGBoost classifiers built on top of UMAP visualisations.......................4

Supplementary Figures for the subsection “Analysis of the Applicability Domains of the models”

Tanimoto similarity distributions for the compounds from the test set to the nearest member of the training set for eTRANSAFE and Drugbank data

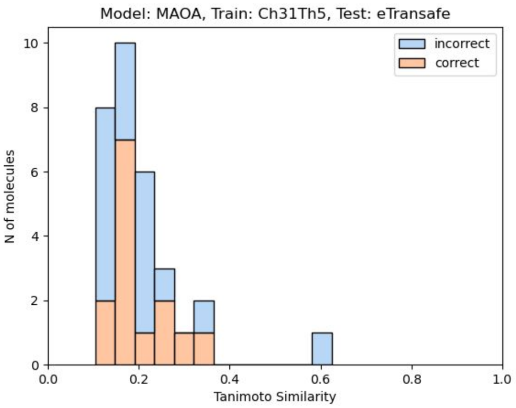

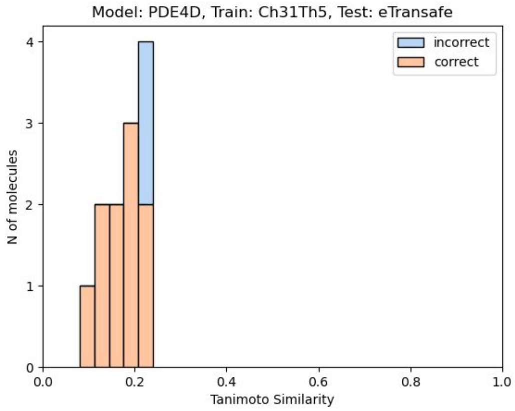

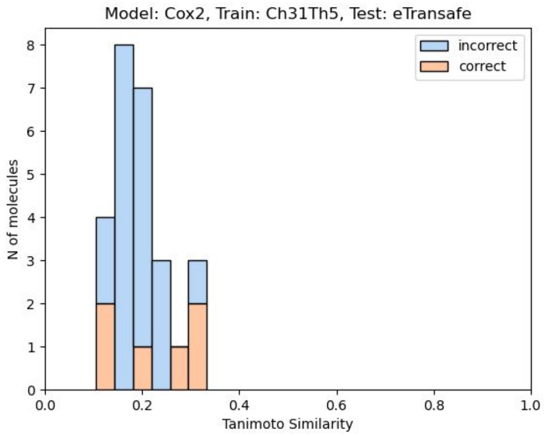

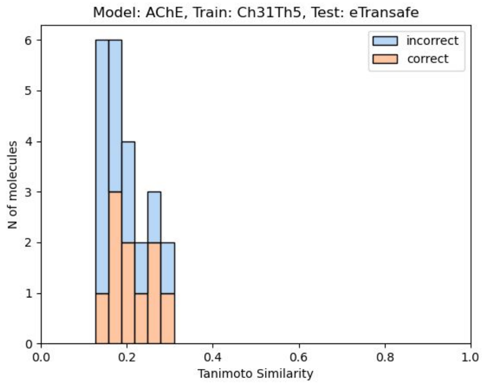

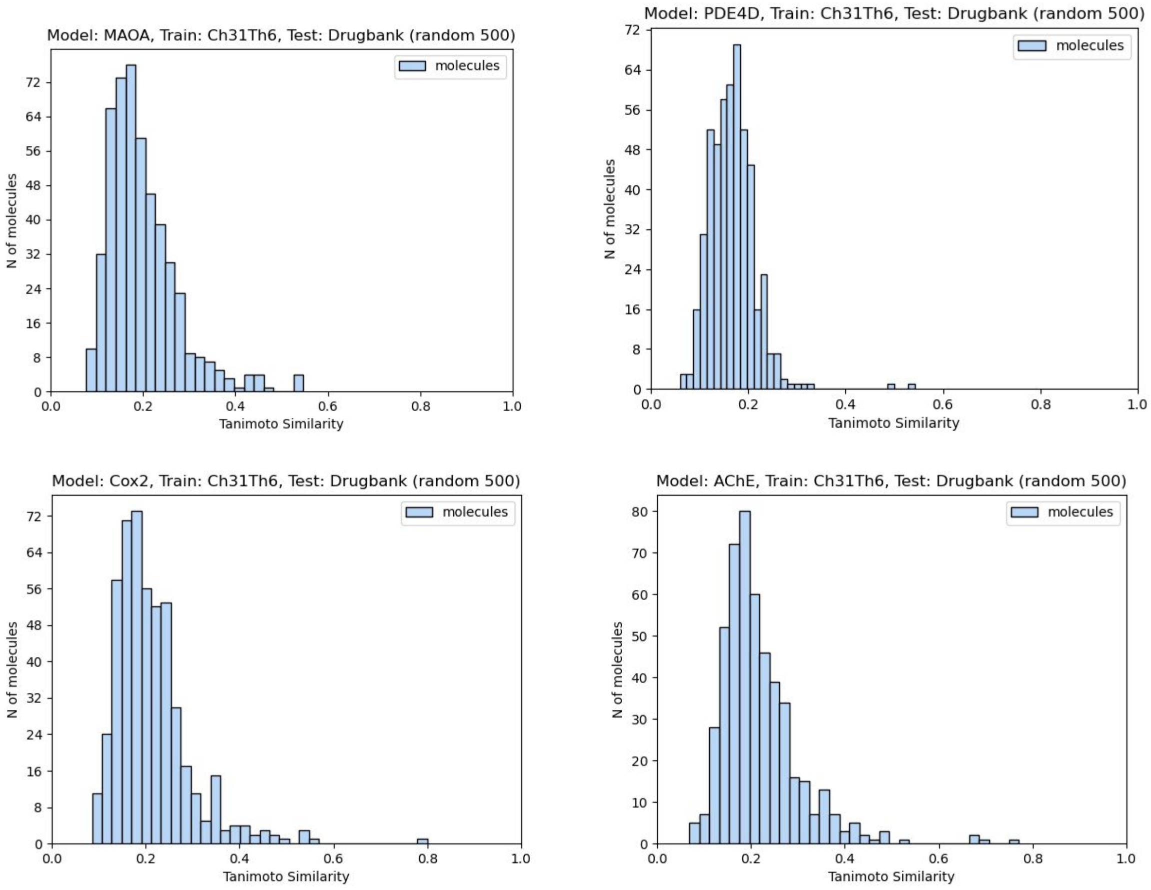

Figure S1. The Tanimoto similarity distributions for the compounds from the test set to the nearest member of the training set for eTRANSAFE and Drugbank data. Blue – incorrect predictions, Red – correct predictions.

Supplementary Tables for the subsection “Results - Model Performance on Publicly Available and Pharmaceutical Test Sets”

Statistical metric for all four off-targets predicted on the eTRANSAFE:

Off-target									
	BA	ACC	Recall	PRC	Sen	Spe	F-score	AUC	MCC
AChE	-	0.43	-	0	-	0.43	-	-	-
MAO-A	0.23	0.45	0	0	0	0.47	-	0.20	-0.19
COX-2	0.28	0.23	0.33	0.05	0.33	0.22	0.09	0.33	-0.32
PDE4D	0.75	0.83	1.00	0.8	1.00	0.50	0.89	0.66	0.63

Table S1: Statistical metric for all four off-targets predicted on the eTRANSAFE data using the ChEMBL31 based model with threshold pChEMBL 5.

Off-target									
	BA	ACC	Recall	PRC	Sen	Spe	F-score	AUC	MCC
AChE	-	0.96	-	-	-	0.96	-	-	-
MAO-A	1.00	1.00	1.00	1.00	1.00	1.00	1.00	1.00	1.00
COX-2	0.62	0.85	0.33	0.33	0.33	0.91	0.33	0.75	0.25
PDE4D	0.50	0.33	0	-	0	1	-	0.47	-

Table S2: Statistical metric for all four off-targets predicted on the eTRANSAFE data by the Naga et al. industry data based models.

ROC AUC performance of the XGBoost classifiers built on top of UMAP visualisations

Model	Endpoint	Type	AUC ROC	
			Mean	Std
ChEMBL	AChE	normal	0.67	0.01
		permutated	0.51	0.01
	Cox2	normal	0.66	0.01
		permutated	0.51	0.01
	MAOA	normal	0.68	0.01
		permutated	0.5	0.02
	PDE4D	normal	0.69	0.01
		permutated	0.51	0.00
Naga et al.	AChE	normal	0.62	0.01

		permutated	0.05	0.01
	Cox2	normal	0.65	0.01
		permutated	0.51	0.01
	MAOA	normal	0.69	0.01
		permutated	0.51	0.01
	PDE4D	normal	0.65	0.02
		permutated	0.51	0.01

Table S3. The ROC AUC performance of the XGBoost classifiers built on top of UMAP visualisations.
